# HBP1 promoter methylation augments the oncogenic β-catenin to correlate with prognosis in NSCLC

**DOI:** 10.1111/jcmm.12318

**Published:** 2014-06-04

**Authors:** Ruo-Chia Tseng, Way-Ren Huang, Su-Feng Lin, Pei-Chen Wu, Han-Shui Hsu, Yi-Ching Wang

**Affiliations:** a Department of Molecular Biology and Human Genetics, College of Life Science, Tzu Chi UniversityHualien, Taiwan; b Department of Biomedical Engineering, School of Biomedical Science and Engineering, National Yang-Ming UniversityTaipei, Taiwan; c Department of Life Sciences, National Taiwan Normal UniversityTaipei, Taiwan; d Division of Thoracic Surgery, Taipei Veterans General HospitalTaipei, Taiwan; e Institute of Emergency and Critical Care Medicine, National Yang-Ming University School of MedicineTaipei, Taiwan; f Department of Pharmacology and Institute of Basic Medical Sciences, College of Medicine, National Cheng Kung UniversityTainan, Taiwan

**Keywords:** HBP1, β-catenin, transcriptional repressor, prognosis, NSCLC

## Abstract

β-catenin nuclear accumulation is frequently identified in human non-small cell lung cancer (NSCLC). The HMG-box transcription factor 1 (HBP1) is a known repressor of β-catenin transactivation. However, the role of HBP1 in relation to β-catenin nuclear accumulation has not been addressed in human cancer patients. In addition, the mechanism of *HBP1* gene alteration in NSCLC remains unclear, although *HBP1* mutation and gene deletion of *HBP1* are reported in breast and colon cancers. Here, we demonstrate that HBP1 acts as a tumour suppressor and serves as a prognostic biomarker in NSCLC clinical and cell models. The immunohistochemistry data indicated that 30.5% (25/82) of tumours from NSCLC patients showed absence or low expression of HBP1 protein. A significant inverse correlation between mRNA/protein expression and promoter hypermethylation suggested that promoter hypermethylation is responsible for low expression of HBP1 in NSCLC patients. Reactivation of HBP1 expression by demethylation reagent or ectopic expression of HBP1 suppressed β-catenin transactivation. Conversely, HBP1 knockdown increased β-catenin transactivation. Importantly, preserved expression of HBP1 had a significantly protective effect on prognosis in patients with β-catenin nuclear accumulation, suggesting that low expression of HBP1 in NSCLC patients with β-catenin nuclear accumulation was one of the major determinants of prognosis. Our data from cellular and clinical models suggest that HBP1 is a suppressor of cancer progression, making it a potential prognostic predictor and therapeutic target to attenuate lung cancer progression.

## Introduction

Lung cancer is one of the most common malignancies in the world and is the leading cause of cancer deaths in industrial countries [1]. Despite the significant improvement in both diagnostic and therapeutic modalities for the treatment of cancer patients, outcome remains poor when the disease has spread to regional lymphatics [2]. Cancer cells have been characterized by multiple changes that affect tumour cells *via* various receptors and subsequent signalling pathways [3]. Therefore, further elucidation of the cellular signalling mechanisms in lung tumorigenesis is important to develop early diagnostic and new effective therapeutic targets.

Mounting evidence suggests that cancer may result from the aberrant activation of normally controlled developmental pathways [4]. Wnt/β-catenin signalling has a dual role in vertebrate development and tumorigenesis [5]. In normal and non-stimulated cells, because of the rapid turnover of β-catenin promoted by the destruction complex, majority of β-catenin protein is present in the adherens junctions with very little in cytoplasmic or nuclear fractions [6]. Activated Wnt signalling inhibits phosphorylation of β-catenin, thereby causing β-catenin to dissociate from the destruction complex and preventing its degradation. The hallmark of Wnt signalling is cytoplasmic accumulation of β-catenin protein; the stabilized β-catenin enters the nucleus where it interacts with the T Cell Factor (TCF) and thereby activates the transcription of downstream target genes such as *cyclin D1* and *c-MYC* [7,8]. Within the cell nucleus, the activity of β-catenin/TCF complex can be inhibited by the HMG-box transcription factor 1 (HBP1), which blocks β-catenin signalling by competing with TCF to bind with β-catenin for transcription of target genes in the Wnt/β-catenin pathway [9]. Over time, Wnt/β-catenin signalling has become a crucial pathway in non-small cell lung cancer (NSCLC) as previously demonstrated in many cancers [5,10,11]. Most studies have focused on the negative regulation of β-catenin accumulation in the suppression of tumorigenesis. However, transcriptional repression could also be an important mechanism for the inhibition of gene expression that is activated by stabilized β-catenin [9]. Therefore, HBP1 transcriptional repressor may be an effective way of blocking the expression of β-catenin target genes.

HBP1 may function as a tumour suppressor by inhibiting the Wnt/β-catenin signalling to block the oncogenic phenotype [9]. In addition, HBP1 is revealed to be essential for oncogene-mediated premature senescence that is lost during malignant transformation [12]. A previous study demonstrated that over half of the tested breast cancer samples (15 of 22) had reduced *HBP1* mRNA levels and *HBP1* mutations/variants were associated with invasive breast cancer [13]. Another study reported 18 out of 22 prostate cancer tissue samples showed reduced *HBP1* mRNA levels [14]. In addition, *HBP1* gene lies within the chromosome 7q31.1 that is frequently deleted in many tumours, such as colon cancer, breast cancer and myeloid leukaemia [15–17]. However, the role of HBP1 in relation to β-catenin nuclear accumulation has not been addressed in human cancer patients.

Our previous study showed that low expression of AXIN2/βTrCP in the degradation complex leads to β-catenin nuclear accumulation in NSCLC patients [11]. We also noticed that among the patients with β-catenin accumulation, some showed association with better survival outcome [18,19], suggesting that antagonists of β-catenin transactivation may exert a protective role in patient outcome. This result prompted us to hypothesize that HBP1 may play an important role in the repression of β-catenin-mediated oncogenic effects in cell nucleus. To address this issue, we performed a comprehensive molecular analysis of the effects of HBP1 alterations on DNA methylation and mRNA/protein expression in clinical and cellular models. The mechanism of HBP1 alteration and its effect on β-catenin signalling in human NSCLC were investigated.

## Materials and methods

### Subjects

Paired tumour and normal lung tissues were obtained from 82 NSCLC patients prospectively recruited at the Taipei Veterans General Hospital between 2002 and 2008 after appropriate institutional review board permission and informed consent from patients were obtained. Overall survival was calculated from the day of surgery to the date of death or the last follow-up. For methylation assay, genomic DNA from primary tumour tissues was extracted by using proteinase K digestion followed by phenol–chloroform extraction. For RNA expression assay, total RNA was extracted from paired tumour and normal tissues by using Trizol reagent (Invitrogen, Carlsbad, CA, USA). cDNA was synthesized by using SuperScriptTM reverse transcriptase (Invitrogen) according to the manufacturer's instructions.

### Immunohistochemical analysis

Paraffin blocks of tumours were sectioned and processed by using standard techniques. Polyclonal antibodies against β-catenin (1:1500; Transduction Laboratories, Lexington, KY, USA), HBP1 (1:200; Santa Cruz Biotechnology, Santa Cruz, CA, USA) and β-actin (1:5000; Abcam, Cambridge, MA, USA) were used as the primary antibodies to detect protein expression. Staining ‘percentage’ for HBP1 was scored as 3, 2, 1, or 0 if >75%, 50–75%, 25–50%, or <25% area were stained, respectively. A score of 1 or 0 indicated the presence of little or no HBP1 protein expression. β-catenin nuclear expression was scored as 3, 2, or 1 if >70%, 36–70%, or 0–35% area were stained, respectively. A score of 3 indicated β-catenin nuclear accumulation.

### Western blot analysis

Cells were lysed and lysates were centrifuged. SDS gel loading buffer (60 mM Tris base, 2% SDS, 10% glycerol and 5% β-mercaptoethanol) was added and samples containing 50 μg of protein were separated on an 8% SDS-PAGE then electro-blotted onto Immobilon-P membranes (Millipore, Bedford, MA, USA). Immunoblotting was performed with antibodies against HBP1 (1:800; Santa Cruz Biotechnology). β-actin (1:5000; Abcam) was used as loading control.

### Quantitative reverse-transcriptase PCR (RT-qPCR) assays

RT-qPCR analysis of clinical samples for *HBP1* and *cyclin D1* mRNA expression was performed with *GAPDH* gene as an internal control, while RT-qPCR of cell models for *HBP1*, *c-myc* and *cyclin D1* mRNA expression was performed with *β-actin* gene as internal control. Primers used are listed in Table S1. The tumour samples with *HPB1* expression levels <50% of corresponding normal tissues were deemed to have an abnormal pattern. The mRNA level of *HBP1*, *c-MYC* and *cyclin D1* were calculated using 2^−ΔCt^ (ΔCt = C_target gene_ − Ct_internal control_).

### Methylation-specific PCR (MSP) assay

The methylation status in the promoter region of the *HBP1* gene was determined by chemical treatment with sodium bisulphite and subsequent MSP analysis. Positive control samples with unmethylated DNA from IMR90 normal lung cell (U reaction) and *Sss*I methyltransferase-treated methylated DNA (M reaction) were included in each PCR set. Primers used are listed in Table S1. Bisulfite-modified DNA (100 ng) was amplified by PCR (35 cycles for U reaction; 45 cycles for M reaction) with annealing temperatures of 55°C and 65°C for U and M reactions, respectively. Hypermethylated genes were defined as those which produced more amplicons of M products than of U products from the samples.

### 5-aza-2′-deoxycytidine (5-Aza-dC) treatment

A549 cells (1 × 10^5^ per dish) were plated in 100-mm culture dish on the day before treatment. The cells were treated with 20 μmol/l 5-Aza-dC (Sigma-Aldrich, St Louis, MO, USA) for three doubling times and then harvested for MSP, RT-qPCR and Western blot.

### Knockdown or ectopic expression

The siRNA-*HBP1*/control siRNA and pCMV-SPORT6-*HBP1*/pCMV-SPORT6 were obtained from OriGene (OriGene Technologies, Rockville, MD, USA) and TransOmics (TransOmics, Huntsville, AL, USA), respectively. A549 cells (1 × 10^5^) were transfected with 5 μg of siRNA-*HBP1* or pCMV-SPORT6-*HBP1* by using ExGen 500 transfection reagent (Fermentas, Pittsburgh, PA, USA) as recommended by the manufacturer. After incubation, cells were subjected to RT-qPCR and Western blot analysis.

### Luciferase reporter assay

The TCF luciferase constructs, containing the wild-type (pTOPFLASH) or mutant (pFOPFLASH) TCF binding sites (Upstate, Lake Placid, NY, USA), were co-transfected with an internal control (pRLTK Renilla luciferase vector; Promega, Madison, WI, USA) into A549/pCMV-SPORT6-*HBP1* and A549/si-*HBP1* cells (5 × 10^5^). The firefly (TOPFLASH or FOPFLASH) luciferase activity was normalized against the Renilla luciferase activity. TOPFLASH activity was also normalized against the FOPFLASH activity.

### Statistical analysis

Pearson's chi-sqaured test was used to compare frequency of protein alteration in NSCLC patients at different disease stages. Overall survival curves were calculated according to the Kaplan–Meier method, and comparison was performed with the log-rank test. Univariate and multivariate analyses were estimated by using Cox proportional hazards regression. *P* < 0.05 was considered statistically significant. SPSS version 19.0 (IBM, Chicago, IL, USA) was used for all statistical analyses.

## Results

### Low HBP1 protein expression correlates with poor prognosis of NSCLC patients

**Fig. 1:**
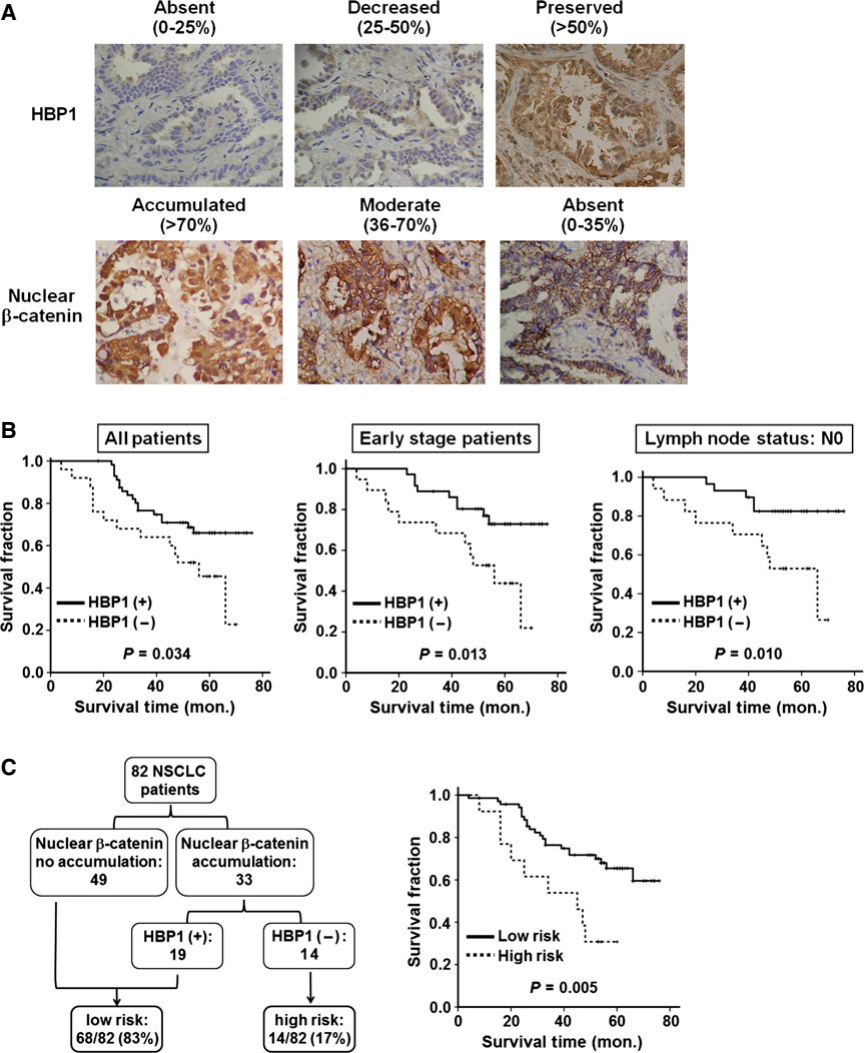
Prognostic analyses of HBP1 protein expression in relation to β-catenin nuclear accumulation. (**A**) Immunohistochemical analysis of HBP1 and β-catenin protein in sections of formalin-fixed, paraffin-embedded tissues from representative NSCLC specimens. ‘Absent or decreased’ indicates low expression of HBP1. ‘Accumulated’ indicates β-catenin nuclear accumulation. Evaluation criteria are described in Materials and Methods. Original magnification ×200. (**B**) Kaplan–Meier survival curves with respect to low protein expression of HBP1. The graphs show the overall, in early stage patients, and in lymph node status (N0) survival of patients by HBP1 expression. (**C**) The 82 NSCLC patients were classified according to risk-of-death categories (left panel) and the Kaplan–Meier estimates of overall survival according to the risk categories (right panel). *P*-value for each analysis is as indicated.

To examine whether HBP1 is altered and associated with the clinical characteristics of the NSCLC patients, we first examined the protein expression level of HBP1 and β-catenin in 82 tumours from NSCLC patient by immunohistochemical analysis (Fig. 1A). The data indicated that 30.5% (25/82) of tumours showed reduced or absent expression of HBP1 while β-catenin nuclear accumulation was found in 40.2% (33/82) of NSCLC, respectively (Table S2). Low HBP1 protein expression correlated with squamous cell carcinoma patients (*P* = 0.046; Table S2). Next, we evaluated the prognostic effects of low HBP1 protein expression by using the Kaplan–Meier method. Unadjusted analysis showed that lower level of HBP1 protein was associated with poor survival in all patients (*P* = 0.034) and patients in early stage (*P* = 0.013) or in lymph node status (N0; *P* = 0.010; Fig. 1B).

### Low HBP1 augments the prognostic effects of β-catenin nuclear accumulation in NSCLC patients

To test our hypothesis that HBP1 exerts a protective role on survival outcome of patients with β-catenin nuclear accumulation, we performed univariate (HBP1 expression, β-catenin nuclear accumulation, smoking habit, tumour type, tumour stage and lymph node status) and multivariate Cox regression analysis. Our data showed that preserved HBP1 expression and lack of β-catenin nuclear accumulation had beneficial effects on prognosis (*P* = 0.039; hazard ratio [HR], 0.48; 95% confidence interval [CI], 0.24–0.96 for preserved HBP1 and *P* = 0.049; HR, 0.38; 95% CI, 0.24–0.99 for no β-catenin nuclear accumulation), even after adjusting for smoking habit, tumour type, tumour stage and lymph node status (*P* = 0.042 for preserved HBP1; *P* = 0.041 for no β-catenin nuclear accumulation; Table 1). In stratification analyses according to the status of β-catenin, preserved HBP1 expression, but not other factors examined, had significantly better effects on prognosis in patients with β-catenin nuclear accumulation (*P* = 0.007; HR, 0.15; 95% CI, 0.04–0.60; Table 2).

**Table 1:** Univariate and multivariate analysis by a Cox regression model

Characteristics	Univariate analysis			Multivariate analysis		
		
	HR (95% CI)	*P*-value*	HR (95% CI)	*P*-value*
HBP1 IHC				
Absent/decreased	1.00		1.00	
Preserved	0.48 (0.24–0.96)	**0.039**	0.38 (0.15–0.96)	**0.042**
β-catenin nuclear accumulation				
Yes	1.00		1.00	
No	0.49 (0.24–0.99)	**0.047**	0.38 (0.15–0.96)	**0.041**
Smoking habit				
Non-smoker	1.00		1.00	
Smoker	0.66 (0.28–1.57)	0.348	0.58 (0.20–1.72)	0.326
Tumour type				
ADC	1.00		1.00	
SCC	1.01 (0.73–2.17)	0.973	1.17 (0.38–3.67)	0.784
Tumour stage				
Early (I/II)	1.00		1.00	
Late (III/IV)	1.58 (0.77–3.26)	0.213	0.68 (0.21–2.26)	0.532
Lymph node status				
N0	1.00		1.00	
≥N1	2.01 (0.99–4.10)	**0.055**	3.34 (1.02–10.92)	**0.046**

* Bold values indicate statistical significance (*P* < 0.05).

CI: confidence interval; HR: hazard ratio; IHC: immunohistochemistry; ADC: adenocarcinoma; SCC: squamous cell carcinoma.

**Table 2:** Hazard ratio for overall survival in the β-catenin group

Characteristics	HR (95% CI)	*P*-value*
β-catenin nuclear accumulation		
HBP1 IHC		
Negative	1.00	
Positive	0.15 (0.04–0.60)	**0.007**
Tumour type		
AD	1.00	
SCC	0.98 (0.30–3.20)	0.970
Tumour stage		
Early (I/II)	1.00	
Late (III/IV)	1.09 (0.14–8.65)	0.937
Lymph node status		
N0	1.00	
≥N1	2.20 (0.24–20.11)	0.484
β-catenin no nuclear accumulation		
HBP1 IHC		
Negative	1.00	
Positive	0.48 (0.14–1.59)	0.228

* Bold values indicate statistical significance (*P* < 0.05).

CI: confidence interval; HR: hazard ratio; IHC: immunohistochemistry; ADC: adenocarcinoma; SCC: squamous cell carcinoma.

Low expression of HBP1 and β-catenin nuclear accumulation were apparently the major determinants of prognosis in NSCLC patients. Therefore, we classified the risk of death among NSCLC patients into two categories. The patients who had either no β-catenin nuclear accumulation or with preserved expression of HBP1 (68/82) were at low risk, whereas those having both β-catenin nuclear accumulation and low HBP1 expression (14/82) were at high risk (Fig. 1C, left panel). Interestingly, Kaplan–Meier analysis showed that NSCLC patients at high risk correlated with worse prognosis (*P* = 0.005; Fig. 1C, right panel). Together, these data suggested that low expression of HBP1 is a clinically relevant regulator of NSCLC that eventually leads to poor prognosis.

### Promoter hypermethylation is the predominant mechanism of low HBP1 expression and enhanced β-catenin activity in NSCLC clinical and cell models

**Fig. 2:**
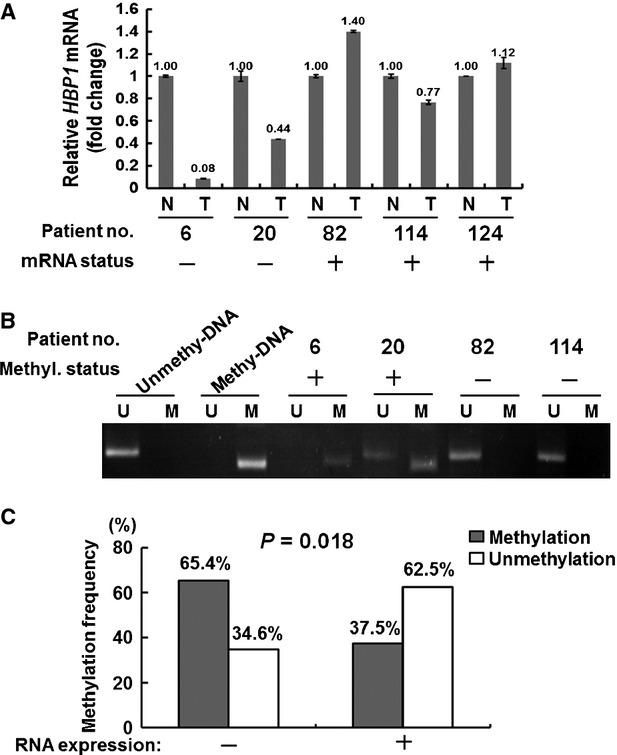
Correlation of mRNA expression and DNA methylation of *HBP1* gene in NSCLC patients. (**A**) RT-qPCR and (**B**) MSP were conducted to analyse *HBP1* mRNA expression and promoter methylation status, respectively. +: positive, −: negative status. N: corresponding normal, T: tumour tissue. The primer sets used for amplification are designated ‘U’ for unmethylated or ‘M’ for methylated genes. (**C**) Concordance analysis between mRNA and promoter methylation for the *HBP1* gene. *P*-value for each analysis is as indicated.

To verify the mechanism involved in altered HBP1 protein expression, we carried out mRNA expression and DNA methylation assays of *HBP1* gene in the cohort of 82 NSCLC patients. Quantitative RT–PCR (RT-qPCR) analysis demonstrated a decrease or absence of *HBP1* transcripts in 31.7% (26/82) of tumour tissues compared to their corresponding normal tissues (Fig. 2A and Table S3). Methylation-specific PCR assay showed that 53.7% (44/82) of tumours exhibited promoter hypermethylation of *HBP1* gene (Fig. 2B and Table S3). Notably, promoter hypermethylation was significantly associated with low mRNA expression (*P* = 0.018; Fig. 2C) and low protein expression (*P* = 0.009; Table S3), suggesting that promoter hypermethylation is responsible for low expression of *HBP1* gene. Next, we evaluated whether epigenetic silencing of *HBP1* led to increased expression of β-catenin-targeted genes, such as *cyclin D1*, in NSCLC patients. We detected the *cyclin D1* mRNA by RT-qPCR. The correlation analysis indicated that high expression of *cyclin D1 in* patients were frequently accompanied by low expression of HBP1 (*P* = 0.042; Fig. S1).

**Fig. 3:**
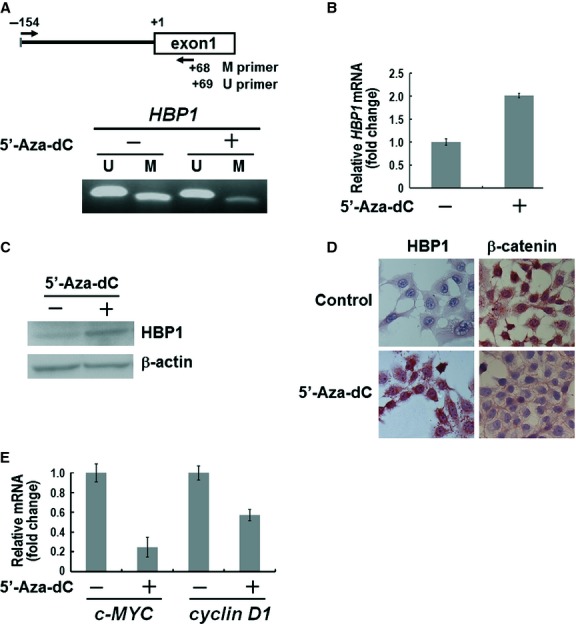
Effect of 5-Aza-dC treatment in A549 lung cancer cells. (**A**) A schematic representation of the genomic structure of *HBP1* locus with the position of the primers used for the MSP assay (upper panel). MSP analysis of *HBP1* gene in A549 cells after 5′-Aza-dC treatment (lower panel). (**B**) RT-qPCR and (**C**) Western blot analysis of HBP1 mRNA and protein re-expression in 5-Aza-dC treated A549 cells. (**D**) Protein expression of HBP1 and β-catenin was measured by immunohistochemical staining in A549 cells treated with or without 5′-Aza-dC. (**E**) RT-qPCR analysis of target genes of β-catenin transactivation, *i.e*., *c-MYC* and *cyclin D1* in A549 cells after 5-Aza-dC treatment. Data are representative of three independent experiments.

To confirm whether promoter hypermethylation of *HBP1* resulted in decreased *HBP1* gene expression and increased β-catenin activity, A549 cells were treated with the demethylating agent 5-Aza-dC and interrogated for the expression of HBP1 and β-catenin transactivation targets *c-MYC* and *cyclin D1*. As shown in Figure 3A–D, treatment with 5-Aza-dC successfully restored mRNA and protein expressions and demethylation of the *HBP1* promoter which was originally methylated in A549 cells. In addition, the immunohistochemical analysis showed that accumulation of nuclear β-catenin was reduced in A549 cells treated with 5′-Aza-dC (Fig. 3D, right panel). Importantly, mRNA expression of *c-MYC* and *cyclin D1* was decreased after restoration of HBP1 expression (Fig. 3E). Our data suggested that promoter hypermethylation of *HBP1* gene could be responsible for low expression and loss of transcriptional repressive activity of HBP1 in NSCLC.

### Ectopically expressed or knocked down HBP1 influences transactivation of β-catenin in NSCLC cell line

**Fig. 4:**
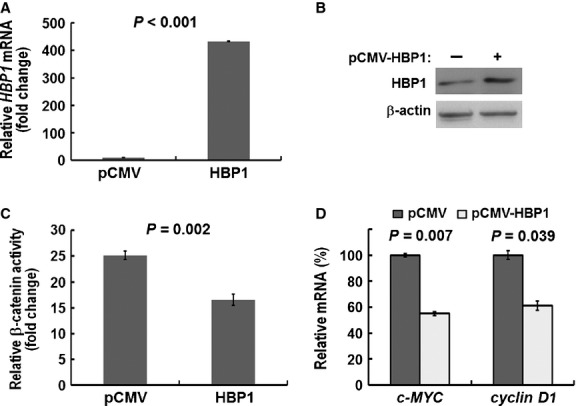
Effect of ectopic expression of HBP1 on β-catenin transactivation in A549 cells. (**A**) RT-qPCR analysis and (**B**) Western blot analysis of HBP1 re-expression by ectopic expression of HBP1 in A549 cells. (**C**) Decreased β-catenin transactivation in A549 cells ectopically expressing HBP1. Cells expressing empty pCMV vector or HBP1 were transfected with the TOPFLASH reporter. (**D**) RT-qPCR analysis of the *c-MYC* and *cyclin D1* mRNA level in cells ectopically expressing HBP1. *P*-value for each analysis is as indicated. Data represent mean ± SD from three independent experiments.

To verify the role of HBP1 in transcriptional regulation of β-catenin/TCF in lung cancer, we examined the effect of ectopically expressed HBP1 in A549 cells. By using RT-qPCR and Western blot assays we confirmed that HBP1 mRNA and protein were increased in cells ectopically expressing HBP1 compared with vector control cells (Fig. 4A and B). We further investigated whether HBP1 expression had effect on the transcriptional activity of β-catenin/TCF by using a TCF reporter/LEF reporter assay (TOPFLASH). The FOPFLASH reporter with mutated LEF/TCF site was used as a negative control. The β-catenin activity was significantly inhibited by ectopically expressed HBP1 (*P* = 0.002; Fig. 4C), whereas there was no change in the negative control FOPFLASH reporter (data not shown). Indeed, the expression of *c-MYC* and *cyclin D1* were decreased in ectopically expressed HBP1 cells (*P* = 0.007 and *P* = 0.039, respectively; Fig. 4D).

**Fig. 5:**
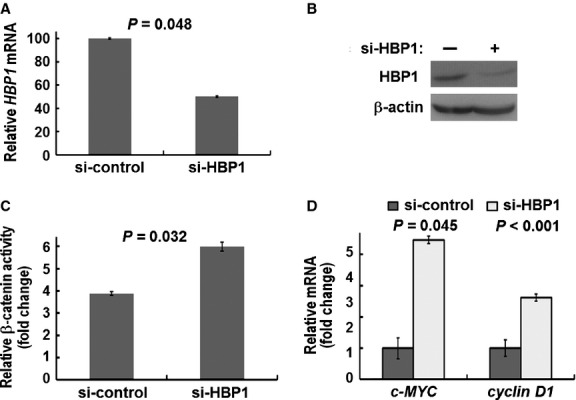
Inverse correlation of HBP1 expression with β-catenin transactivation by using HBP1 knockdown in A549 cells. (**A**) RT-qPCR analysis and (**B**) Western blot analysis of siRNA-HBP1-induced gene knockdown in A549 cells. (**C**) Increased β-catenin transactivation in A549 cells after HBP1 knockdown. Control siRNA and siRNA-HBP1 cells were transfected with the TOPFLASH reporter. (**D**) RT-qPCR analysis of the *c-MYC* and *cyclin D1* mRNA level in cells after HBP1 knockdown. *P*-value for each analysis is as indicated. Data represent mean ± SD from three independent experiments.

To further confirm the reciprocal relationship between HBP1 expression and β-catenin activity in lung cancer, we examined whether HBP1 knockdown would affect β-catenin/TCF activity and increase *c-MYC* and *cyclin D1* expression. A549 cells transfected with siRNA-*HBP1* showed low HBP1 mRNA and protein expression compared with control siRNA (Fig. 5A and B). β-catenin activity was significantly increased in HBP1 knockdown A549 cells (*P* = 0.032; Fig. 5C). In addition, the expression of *c-MYC* and *cyclin D1* were induced in HBP1 knockdown A549 cells (*P* = 0.045 and, *P* < 0.001, respectively; Fig. 5D). Altogether, our data suggested that HBP1 expression plays an important role in suppressing β-catenin transactivation in lung cancer.

## Discussion

**Fig. 6:**
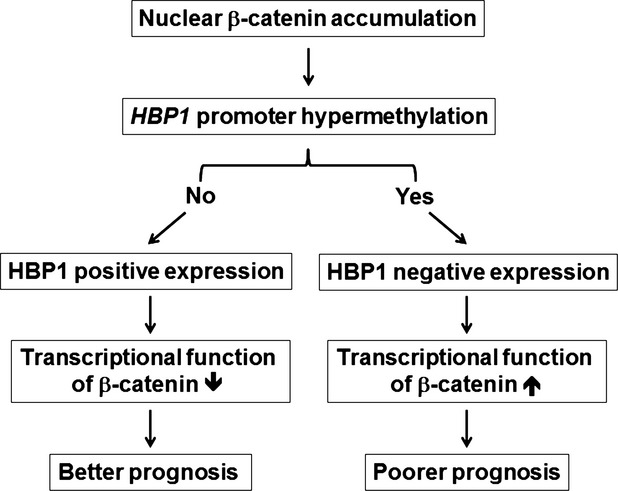
The model showing relationship of HBP1 alteration and β-catenin nuclear accumulation with prognostic effect in NSCLC patients. HBP1 is one of the major determinants of prognosis in NSCLC patients with β-catenin nuclear accumulation. Patients with *HBP1* gene silencing by promoter hypermethylation and low activity in suppressing β-catenin transactivation show poorer prognosis compared to those without *HBP1* promoter hypermethylation.

In an effort to better understand the mechanism of HBP1 alteration in NSCLC patients, we carried out a comprehensive molecular analysis including mRNA/protein expression and promoter methylation of *HBP1* gene in 82 NSCLC patients. Our study unravels a new mechanism involved in HBP1 gene silencing by promoter hypermethylation and low activity in suppressing β-catenin transactivation. We also evaluated the prognostic effect of HBP1 alteration and β-catenin nuclear accumulation in NSCLC patients. Low expression of HBP1 may be the major determinant of prognosis in NSCLC patients with β-catenin nuclear accumulation (Fig. 6).

The tumour suppressor genes are known to be inactivated by genetic and epigenetic alterations [20]. HBP1 has been previously reported to be inactivated by mutations or gene deletion [13,15–17]. However, our previous data showed that deletion at 7q is not frequently found in NSCLC patients [21], suggesting that other mechanisms may be involved in HBP1 inactivation in NSCLC. In the present study, clinical data suggested that promoter hypermethylation is involved in the deregulation of *HBP1* gene. We further identified that reactivation of HBP1 by DNA demethylation indeed reduced β-catenin nuclear transactivation as evident by the low mRNA expression of several target genes of β-catenin. Our study provides a link for the transactivation of nuclear β-catenin through epigenetic inactivation of HBP1 in lung cancer.

Recently, Pan *et al*. reported that HBP1 transcriptionally represses DNMT1 expression resulting in both gene-specific and global DNA hypomethylation in lung fibroblast cell lines [22]. Therefore, we examined whether low HBP1 expression correlated with promoter hypermethylation of known tumour suppressor genes including *AXIN2* [11], *BTRCP* [11] and *HIC1* [23] in NSCLC patients. Low HBP1 expression was significantly associated with hypermethylation of *AXIN2* and *BTRCP* genes and to a lesser extent with *HIC1* gene (Table S4). These results suggested that HBP1 may participate in regulating DNA methylation in NSCLC cancer patients. Notably, our data showed that the accumulation of nuclear β-catenin was reduced in cells with 5′-Aza-dC treatment (Fig. 3D). The 5′-Aza-dC is a strong inducer of DNA hypomethylation that is well-known to resuscitate epigenetically silenced genes [24]. In our previous study, A549 cells showed promoter hypermethylation and low expression of *AXIN2* gene, which encodes the component of β-catenin degradation complex. 5′-Aza-dC treatment successfully restored AXIN2 mRNA and protein expressions [11]. The reactivation of AXIN2 degradation complex may contribute in part to the substantial decrease in β-catenin protein expression that we observed in cells treated with 5-Aza-dC.

We found that low expression of HBP1 protein was significantly associated with poor survival in all NSLCLC patients. We further performed the prognosis analyses of *HBP1* mRNA expression in publicly available microarray data of NSCLC by the PrognoScan database (http://www.abren.net/PrognoScan/). As shown in Figure S2, the results support our conclusion that low HBP1 expression was associated with poor survival. These clinical data indicate the relevance of our finding which can be also seen in other cohorts of patients as part of publicly available microarray expression data [25,26]. Notably, our Cox regression analysis revealed that low HBP1 expression was the major determinant of prognosis in NSCLC patients with β-catenin nuclear accumulation. These data suggested that low HBP1 expression in patients with β-catenin nuclear accumulation could be a useful prognostic factor in NSCLC.

Our clinical and cell model findings provide evidence that HBP1 expression can down-regulate β-catenin transactivation. Recently, Kim *et al*. found that epigallocatechin 3-gallate, the major phytochemical in green tea, blocks Wnt signalling by inducing the HBP1 transcriptional repressor and inhibits breast cancer tumorigenesis [27]. In addition, treatment with N-acetylcysteine, an anti-cancer compound, suppresses cell growth by increasing the expression of HBP1, but HBP1 knockdown attenuated growth arrest and apoptosis in oral cancer [28]. Therefore, strategies to increase HBP1 expression may be useful for cancer prevention or treatment. The search for antagonists or agonists of the HBP1 may also lead to the discovery of compounds that can potentially be used for lung cancer treatment.

## Supporting information

**Figure S1** (a) Bar graph shows the percentage of expression of different proteins in NSCLC patients. **Figure S2** Low *HBP1* mRNA expression correlates with poor survival of NSCLC patients in the publicly available microarray datasets in (a) jacob-00182-CANDF project 25 and (b) GSE31210 project 26. Overall survival curve of Kaplan-Meier method was performed in patients with preserved (red lines) and low (blue lines) *HBP*1 mRNA expression. P values were determined using log-rank test. **Table S1** List of primer sequences used in the present study. **Table S2** Correlation between HBP1 or β-catenin expression and clinicopathological parameters of lung cancer. **Table S3** Correlation between *HBP1* mRNA expression or promoter methylation and clinicopathological parameters of lung cancer. **Table S4** Correlation between HBP1 expression and promoter methylation of *AXIN2*, *BTRCP* and *HIC1* genes in lung cancer patients.

## References

[1] Parkin DM, Pisani P, Ferlay J (1999). Global cancer statistics. CA Cancer J Clin.

[2] Jemal A, Tiwari RC, Murray T (2004). Cancer statistics, 2004. CA Cancer J Clin.

[3] Reinmuth N, Mesters RM, Bieker R (2004). Signal transduction pathways as novel therapy targets in lung cancer. Lung cancer.

[4] Daniel VC, Peacock CD, Watkins DN (2006). Developmental signalling pathways in lung cancer. Respirology.

[5] Polakis P (2000). Wnt signaling and cancer. Genes Dev.

[6] Chen RH, Ding WV, McCormick F (2000). Wnt signaling to beta-catenin involves two interactive components. Glycogen synthase kinase-3beta inhibition and activation of protein kinase C. J Biol Chem.

[7] He TC, Sparks AB, Rago C (1998). Identification of c-MYC as a target of the APC pathway. Science.

[8] Shtutman M, Zhurinsky J, Simcha I (1999). The cyclin D1 gene is a target of the beta-catenin/LEF-1 pathway. Proc Natl Acad Sci USA.

[9] Sampson EM, Haque ZK, Ku MC (2001). Negative regulation of the Wnt-beta-catenin pathway by the transcriptional repressor HBP1. EMBO J.

[10] Moon RT, Kohn AD, De Ferrari GV (2004). WNT and beta-catenin signalling: diseases and therapies. Nat Rev Genet.

[11] Tseng RC, Lin RK, Wen CK (2008). Epigenetic silencing of AXIN2/betaTrCP and deregulation of p53-mediated control lead to wild-type beta-catenin nuclear accumulation in lung tumorigenesis. Oncogene.

[12] Zhang X, Kim J, Ruthazer R (2006). The HBP1 transcriptional repressor participates in RAS-induced premature senescence. Mol Cell Biol.

[13] Paulson KE, Rieger-Christ K, McDevitt MA (2007). Alterations of the HBP1 transcriptional repressor are associated with invasive breast cancer. Cancer Res.

[14] Chen YC, Zhang XW, Niu XH (2010). Macrophage migration inhibitory factor is a direct target of HBP1-mediated transcriptional repression that is overexpressed in prostate cancer. Oncogene.

[15] Zenklusen JC, Thompson JC, Klein-Szanto AJ (1995). Frequent loss of heterozygosity in human primary squamous cell and colon carcinomas at 7q31.1: evidence for a broad range tumor suppressor gene. Cancer Res.

[16] Driouch K, Briffod M, Bieche I (1998). Location of several putative genes possibly involved in human breast cancer progression. Cancer Res.

[17] Liang H, Fairman J, Claxton DF (1998). Molecular anatomy of chromosome 7q deletions in myeloid neoplasms: evidence for multiple critical loci. Proc Natl Acad Sci USA.

[18] Hommura F, Furuuchi K, Yamazaki K (2002). Increased expression of beta-catenin predicts better prognosis in nonsmall cell lung carcinomas. Cancer.

[19] Chiu CG, Chan SK, Fang ZA (2012). Beta-catenin expression is prognostic of improved non-small cell lung cancer survival. Am J Surg.

[20] Sadikovic B, Al-Romaih K, Squire JA (2008). Cause and consequences of genetic and epigenetic alterations in human cancer. Curr Genomics.

[21] Tseng RC, Chang JW, Hsien FJ (2005). Genomewide loss of heterozygosity and its clinical associations in non small cell lung cancer. Int J Cancer.

[22] Pan K, Chen Y, Roth M (2013). HBP1-mediated transcriptional regulation of DNA methyltransferase 1 and its impact on cell senescence. Mol Cell Biol.

[23] Tseng RC, Lee CC, Hsu HS (2009). Distinct HIC1-SIRT1-p53 loop deregulation in lung squamous carcinoma and adenocarcinoma patients. Neoplasia.

[24] Michalowsky LA, Jones PA (1987). Differential nuclear protein binding to 5-azacytosine-containing DNA as a potential mechanism for 5-aza-2′-deoxycytidine resistance. Mol Cell Biol.

[25] Shedden K, Taylor JM, Director's Challenge Consortium for the Molecular Classification of Lung A (2008). Gene expression-based survival prediction in lung adenocarcinoma: a multi-site, blinded validation study. Nat Med.

[26] Okayama H, Kohno T, Ishii Y (2012). Identification of genes upregulated in ALK-positive and EGFR/KRAS/ALK-negative lung adenocarcinomas. Cancer Res.

[27] Kim J, Zhang X, Rieger-Christ KM (2006). Suppression of Wnt signaling by the green tea compound (-)-epigallocatechin 3-gallate (EGCG) in invasive breast cancer cells. Requirement of the transcriptional repressor HBP1. J Biol Chem.

[28] Lee MF, Chan CY, Hung HC (2013). N-acetylcysteine (NAC) inhibits cell growth by mediating the EGFR/Akt/HMG box-containing protein 1 (HBP1) signaling pathway in invasive oral cancer. Oral Oncol.

