# Supplementary material for: HBP1 promoter methylation augments the oncogenic β-catenin to correlate with prognosis in NSCLC
Source: J Cell Mol Med. 2014 Jun 4;18(9):1752–61. doi: 10.1111/jcmm.12318 (PMC4196651; doi:10.1111/jcmm.12318)
Supplement: Supplementary file 1 [file jcmm0018-1752-SD1.doc]

**Table S1. List of primer sequences used in the present study.**

| Gene | Primer | 5’🡪3’ sequences | PCR size (bp) | Tm (^o^C) | Cycle number |
| --- | --- | --- | --- | --- | --- |
| *HBP1* | Forward | CTTTGTCTTGTGGAGGACCTG | 396 | 60 | 35 |
|  | Reverse | GTGAGCCTGAATTGGTTCTTTT |  |  |  |
| *c-MYC* | Forward | TGAAAGGCTCTCCTTGCAGC | 175 | 58 | 32 |
|  | Reverse | GCTGGTAGAAGTTCTCCTCC |  |  |  |
| *cyclin D1* | Forward | ATGTGTGCAGAAGGAGGTCC | 199 | 61 | 32 |
|  | Reverse | CTTAGAGGCCACGAACATGC |  |  |  |
| *GAPDH* | Forward | AATCCCATCACCATCTTCCA | 588 | 55 | 30 |
|  | Reverse | CCTGCTTCACCACCTTCTTG |  |  |  |
| *β-actin* | Forward | GGCGGCACCACCATGTACCCT | 180 | 58 | 35 |
|  | Reverse | AGG GGCCGGACTCGTCATACT |  |  |  |
| *HBP1*-methyl-M | Forward | TTTTTCGAGACGGATTACGG | 220 | 65 | 45 |
|  | Reverse | GCGAATTACTACTCCCGCGAC |  |  |  |
| *HBP1*-methyl-U | Forward | TTTTTTGAGATGGATTATGG | 223 | 55 | 35 |
|  | Reverse | CACAAATTACTACTCCCACAAC |  |  |  |

**Table S2. Correlation between HBP1 or β-catenin expression and clinicopathological parameters of lung cancer.**

|  |  | **HBP1** | | | | **Nuclear β-catenin** | | | |
| --- | --- | --- | --- | --- | --- | --- | --- | --- | --- |
| Characteristics |  | Total* | + (%) | ─ (%) | *P* value^†^ | Total^*^ | + (%) | ─ (%) | *P* value^†^ |
|  |  |  |  |  |  |  |  |  |  |
| Overall |  | 82 | 57(69.5) | 25(30.5) |  | 82 | 33(40.2) | 49(59.8) |  |
|  |  |  |  |  |  |  |  |  |  |
| Gender | Female | 19 | 13(68.4) | 6(31.6) | 0.906 | 19 | 7(36.8) | 12(23.2) | 0.730 |
|  | Male | 63 | 44(68.8) | 19(30.2) |  | 63 | 26(41.3) | 37(58.7) |  |
|  |  |  |  |  |  |  |  |  |  |
| Smoking habit | Nonsmoker | 12 | 10(83.3) | 2(16.7) | 0.362 | 12 | 4(33.3) | 8(66.7) | 0.555 |
|  | Smoker | 54 | 38(70.4) | 16(29.6) |  | 54 | 23(42.6) | 31(57.4) |  |
|  |  |  |  |  |  |  |  |  |  |
| Tumor type | ADC^‡^ | 49 | 38(77.6) | 11(22,4) | **0.046** | 49 | 20(40.8) | 29(59.2) | 0.520 |
|  | SCC | 27 | 15(55.6) | 12(44.4) |  | 27 | 9(33.3) | 18(66.7) |  |
|  |  |  |  |  |  |  |  |  |  |
| Tumor stage | Early (I/II) | 55 | 36(65.5) | 19(34.5) | 0.255 | 55 | 21(38.2) | 34(61.8) | 0.587 |
|  | Late (III/IV) | 27 | 21(77.8) | 6(22.2) |  | 27 | 12(44.4) | 15(55.6) |  |
|  |  |  |  |  |  |  |  |  |  |

* Total number of sample in some categories is less than the overall number analyzed because clinical data was not available for these samples.

^†^ Bold values indicate statistical significance (*P*<0.05).

^‡^ Abbreviations: ADC, adenocarcinoma; SCC, squamous cell carcinoma.

**Table S3. Correlation between *HBP1* mRNA expression or promoter methylation and clinicopathological parameters of lung cancer.**

|  |  | **mRNA expression** | | | | **Promoter methylation** | | | |
| --- | --- | --- | --- | --- | --- | --- | --- | --- | --- |
| Characteristics |  | Total* | + (%) | ─ (%) | *P* value^†^ | Total* | M^‡^ (%) | U (%) | *P* value^†^ |
|  |  |  |  |  |  |  |  |  |  |
| Overall |  | 82 | 56(68.3) | 26(31.7) |  | 82 | 44(53.7) | 38(46.3) |  |
|  |  |  |  |  |  |  |  |  |  |
| Gender | Female | 19 | 15(78.9) | 4(21.1) | 0.255 | 19 | 13(31.6) | 6(68.4) | 0.141 |
|  | Male | 63 | 41(65.1) | 22(34.9) |  | 63 | 31(50.8) | 32(49.2) |  |
|  |  |  |  |  |  |  |  |  |  |
| Smoking habit | Nonsmoker | 12 | 11(91.7) | 1(8.3) | **0.042** | 12 | 7(58.3) | 5(41.7) | 0.523 |
|  | Smoker | 54 | 33(61.1) | 21(38.9) |  | 54 | 26(48.1) | 28(51.9) |  |
|  |  |  |  |  |  |  |  |  |  |
| Tumor type | ADC^‡^ | 49 | 38(77.6) | 11(22,4) | **0.009** | 49 | 29(59.2) | 20(40.8) | 0.354 |
|  | SCC | 27 | 13(48.1) | 14(51.9) |  | 27 | 13(48.1) | 14(51.9) |  |
|  |  |  |  |  |  |  |  |  |  |
| Tumor stage | Early (I/II) | 55 | 38(69.1) | 17(30.9) | 0.825 | 55 | 27(49.1) | 28(50.9) | 0.236 |
|  | Late (III/IV) | 27 | 18(66.7) | 9(33.3) |  | 27 | 17(63.0) | 10(37.0) |  |
|  |  |  |  |  |  |  |  |  |  |
|  |  |  |  |  |  |  |  |  |  |
| Correlation | protein (+) | 57 | 43(75.4) | 14(24.6) | **0.036** | 57 | 21(36.8) | 36(63.2) | **0.009** |
|  | protein (─) | 25 | 13(52.0) | 12(48.0) |  | 25 | 17(68.0) | 8(32.0) |  |
|  |  |  |  |  |  |  |  |  |  |

* Total number of sample in some categories is less than the overall number analyzed because clinical data was not available for these samples.

^†^ Bold values indicate statistical significance (*P*<0.05).

^‡^ Abbreviations: ADC, adenocarcinoma; SCC, squamous cell carcinoma; M, hypermethylation; U, unmethylation.

**Table S4.** Correlation between HBP1 expression and promoter methylation of *AXIN2*, *BTRCP*, and *HIC1* genes in lung cancer patients.

|  | Methylation | **HBP1** | | | |
| --- | --- | --- | --- | --- | --- |
| Genes | status | Total* | + (%) | ─ (%) | *P* value^†^ |
|  |  |  |  |  |  |
| Overall |  | 82 | 57(69.5) | 25(30.5) |  |
|  |  |  |  |  |  |
| *AXIN2* | M^‡^ | 42 | 23(54.8) | 19(45.2) | **0.005** |
|  | U | 38 | 32(84.2) | 6(15.8) |  |
|  |  |  |  |  |  |
| *BTRCP* | M | 47 | 28(59.6) | 19(40.4) | **0.035** |
|  | U | 33 | 27(81.8) | 6(18.2) |  |
|  |  |  |  |  |  |
| *HIC1* | M | 49 | 16(55.2) | 13(44.8) | 0.067 |
|  | U | 27 | 34(75.6) | 11(24.4) |  |
|  |  |  |  |  |  |

* Total number of sample in some categories is less than the overall number analyzed because clinical sample was not available for these genes.

^†^ Bold values indicate statistical significance (*P*<0.05).

^‡^ Abbreviations: M, hypermethylation; U, unmethylation.


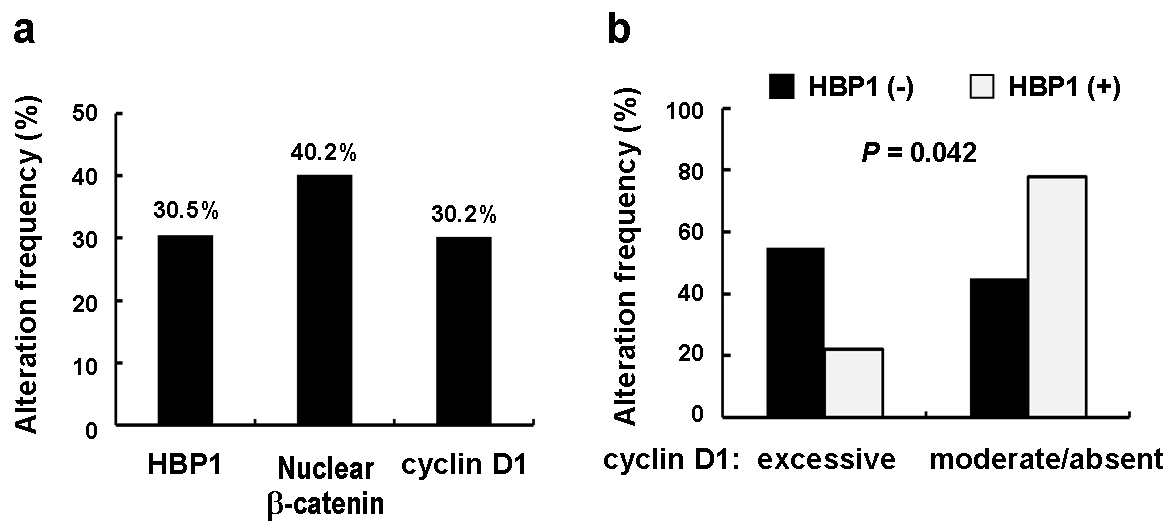


**Figure S1.** (a) Bar graph shows the percentage of expression of different proteins in NSCLC patients. (b) The χ2 analysis shows statistically significant correlation between HBP1 low expression and cyclin D1 overexpression in NSCLC patients.


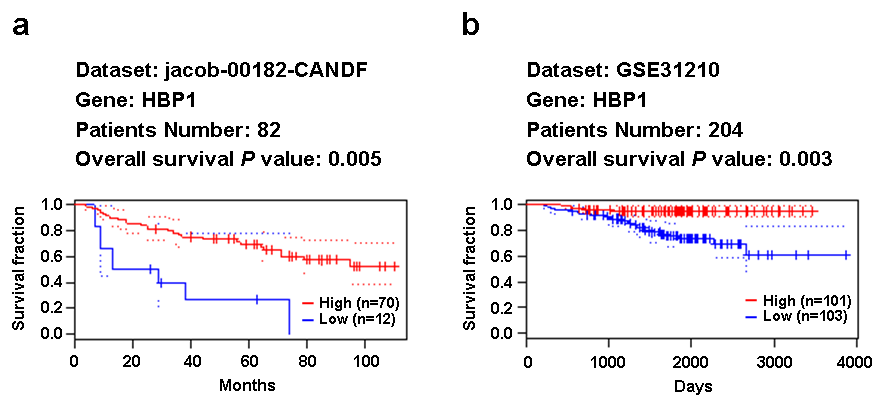


**Figure S2.** Low HBP1 mRNA expression corelates with poor survival of NSCLC patients in the publicly available microarray datasets in (a) jacob-00182-CANDF project [25] and (b) GSE31210 project [26] Overall survival curve of Kaplan-Meier method was performed in patients with preserved (red lines) and low (blue lines) HBP1 mRNA expression. P values were determined using log-rank test.
